# Jimmy Buffett’s death from Merkel cell carcinoma was associated with an increase in online search activity related to skin cancer

**DOI:** 10.1016/j.jdin.2024.03.014

**Published:** 2024-04-02

**Authors:** Tyler B. Cummins, Donald R. Miller, Alan C. Geller

**Affiliations:** a Manchester Essex Regional High School, Manchester, Massachusetts; b Center for Population Health, Department of Biomedical and Nutritional Sciences, University of Massachusetts, Lowell, Massachusetts; c Department of Social and Behavioral Sciences, Harvard TH Chan School of Public Health, Boston, Massachusetts

**Keywords:** celebrity, internet use, melanoma, Merkel cell carcinoma, public health, skin cancer, sun protection

*To the Editor:* Understanding patterns of public interest in skin cancer can help the medical community effectively reach individuals through targeted, well-timed public health education. The influence of celebrity health and disease on internet activity has been demonstrated by spikes in Google search activity for other cancers immediately after their announcements of cancer diagnosis, treatment, or death.^1^^,^^2^

On September 1, 2023, Jimmy Buffett died of Merkel cell carcinoma, a rare but deadly cancer associated with Merkel polyomavirus and sun exposure–induced genetic damage.^3^ Wikipedia pageviews and Google Trends data were evaluated during the 2-week periods before and after Jimmy Buffett’s death to determine whether awareness of his death was associated with increased interest in skin cancers and sun protection.^4^^,^^5^ Google Trends settings were selected as web searches in all categories in the United States.

After Jimmy Buffet’s death, Wikipedia pageviews of the pages “Jimmy Buffett,” “Merkel Cell Carcinoma,” and “Skin Cancer” each sharply increased within 2 days of his death, and each remained substantially elevated for 3 to 7 days. Pageviews for “Melanoma” show a subtle increasing trend. Wikipedia pageviews for sunscreen remained low throughout the study (Fig 1).

**Fig 1:**
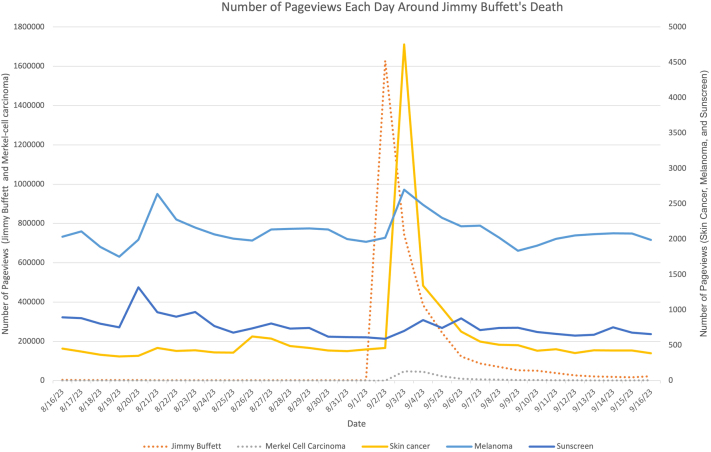
Wikipedia pageviews for Jimmy Buffett, Merkel cell carcinoma, skin cancer, and melanoma increased after the announcement of Jimmy Buffett’s death on September 1, 2023. Daily total pageviews for Jimmy Buffett and Merkel cell carcinoma use the scale shown on the left-hand vertical axis, whereas daily total pageviews for skin cancer, melanoma, and sun protection use the scale shown on the right-hand vertical axis.

Likewise, Google searches for “Merkel Cell Carcinoma” and “Skin Cancer” peaked on September 3, 2023, similar to searches for “Jimmy Buffett.” “Melanoma” searches also trended slightly upward. In contrast, baseline Google search and Wikipedia data from 2022 did not reveal a seasonal peak for these pages and terms in September 2022.

Google relative search volume for Jimmy Buffett from September 2, 2023, to September 15, 2023, shows that this topic was particularly popular in the southeastern United States, possibly due to Jimmy Buffett’s emphasis on sun and beach (Fig 2). This population has a substantial sun exposure and skin cancer burden. Google Trends geographic data from September 2, 2023, to September 22, 2023, demonstrate a correlation between states’ relative search volume for “Jimmy Buffett” and increase in the state’s relative search volume for “Skin Cancer.”

**Fig 2:**
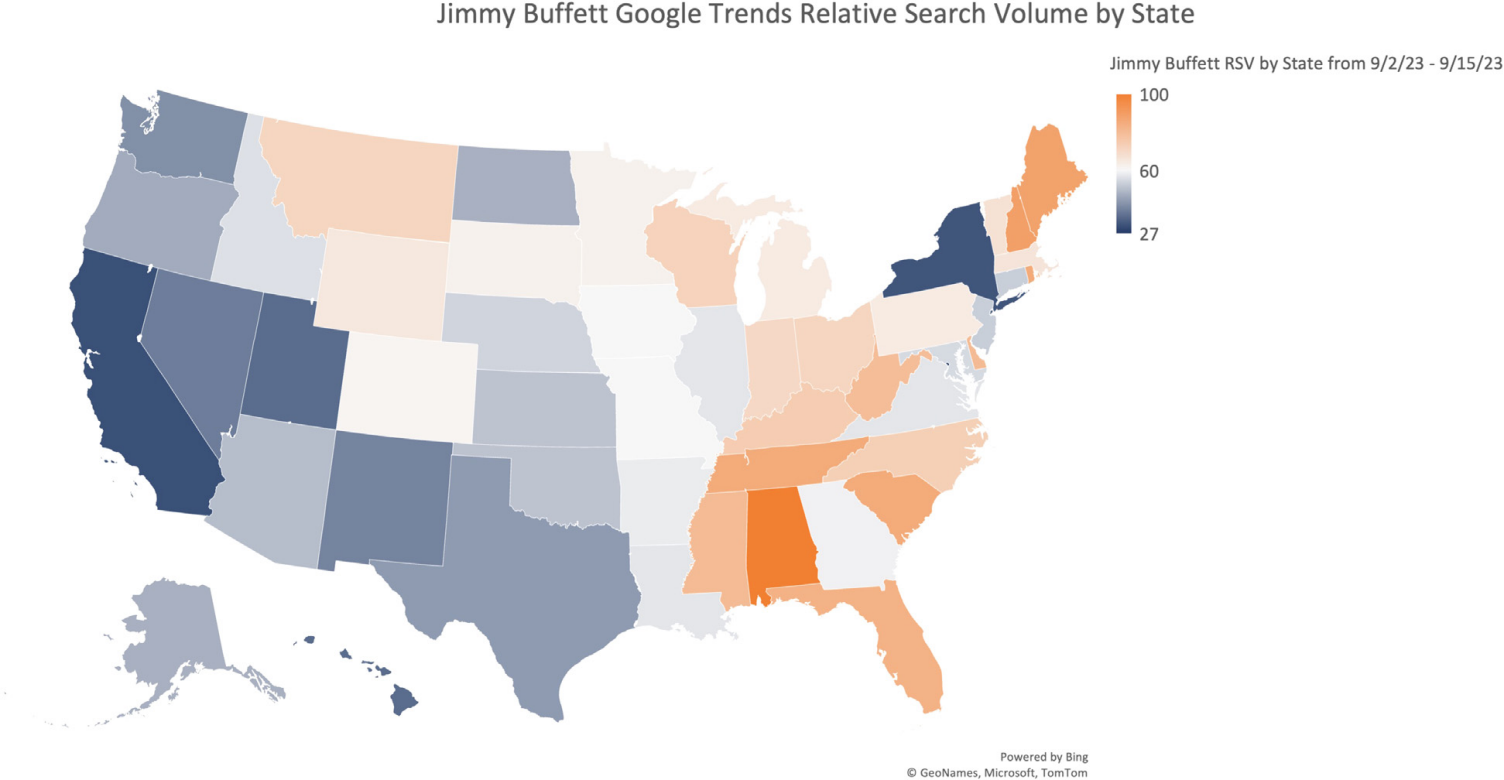
Google relative search volumes for Jimmy Buffett are shown by state. A 3-color gradient is used to visualize geographic variation in searches for “Jimmy Buffett” during the period from September 2, 2023, to September 15, 2023. Many states in the Southeastern United States have higher than average relative search volumes.

Limitations include the relative nature of Google Trends data, although the Wikipedia pageviews data reflect total number of pageviews. Additionally, although a correlation is observed, a causative relationship is not confirmed, and it is unclear whether the increases are the result of news coverage of Jimmy Buffett’s death or whether other factors, such as intentional outreach by skin cancer organizations, contributed to the boost in searches.

These findings suggest an opportunity to provide education on related topics with strong public health impact at a time when many individuals are likely to be curious and receptive to the information. For example, dermatology and other medical societies and other groups, such as the growing number of melanoma foundations dedicated to awareness building, could increase outreach activities, such as social media engagement, blog posting, and potentially targeted advertisements, to provide well-timed education and actionable advice. Although individuals might seek out general information on their own, targeted public health education during the narrow time window of increased interest may more effectively promote behavior modification.

## Conflicts of interest

None disclosed.
